# Temporal trends in initiation of VKA, rivaroxaban, apixaban and dabigatran for the treatment of venous thromboembolism - A Danish nationwide cohort study

**DOI:** 10.1038/s41598-017-03596-x

**Published:** 2017-06-13

**Authors:** Caroline Sindet-Pedersen, Jannik Langtved Pallisgaard, Laila Staerk, Jeffrey S. Berger, Morten Lamberts, Christian Torp-Pedersen, Gunnar H. Gislason, Jonas Bjerring Olesen

**Affiliations:** 1 Department of Cardiology, Copenhagen University Hospital Herlev and Gentofte, 2900 Hellerup, Denmark; 2 Faculty of Health and Medical Sciences, University of Copenhagen, 2200 Copenhagen N, Denmark; 3 New York University School of Medicine, New York, New York USA; 4 Department of Cardiology, Copenhagen University Hospital Rigshospitalet, 2100 Copenhagen Ø, Denmark; 5 Department of Health, Science and Technology, Aalborg University, and Department of Epidemiology/Biostatistics and Cardiology, Aalborg University Hospital, Aalborg, Denmark; 6 The National Institute of Public Health, University of Southern Denmark, 1353 Odense, Denmark; 7 The Danish Heart Foundation, 1127 Copenhagen K, Copenhagen, Denmark

## Abstract

Danish nationwide registries were used to investigate temporal trends in initiation of rivaroxaban or apixaban or dabigatran versus vitamin K antagonists (VKA) in patients with venous thromboembolism (VTE). Patients treated with one of the NOACs (rivaroxaban, dabigatran, apixaban) or VKA were identified between February 2012 and September 2016. A total of 19,578 patients were included of which 10,844 (55.4%) were treated with VKA and 8,734 (44.6%) were treated with NOACs (rivaroxaban 7,572, apixaban 1,066, and dabigatran 96). Temporal trends showed a decrease in the initiation of VKA (p-value for decreasing trend, p < 0001) and an increase in the initiation of rivaroxaban and apixaban (p-value for increasing trend, p < 0001). By September 2016, 12%, 70%, 16%, and 2% of patients with VTE were initiated on VKA, rivaroxaban, apixaban, and dabigatran. Patients with previous VTE, chronic kidney disease, liver disease, cancer, and thrombophilia were more likely to be initiated on VKA compared with one of the NOACs. In conclusion the initiation of rivaroxaban and apixaban is increasing significantly over time in patients with VTE. Patients with previous VTE, chronic kidney disease, liver disease, cancer, and thrombophilia were more likely to be initiated on VKA compared with rivaroxaban or apixaban.

## Introduction

For decades, vitamin K antagonists (VKA) have been the foundation of treatment of venous thromboembolism (VTE)^1, 2^. While VKA is effective in treating VTE, the treatment has several drawbacks, which includes narrow therapeutic interval, need for frequent blood tests, bridging therapy, and interactions with food and drugs^1, 3, 4^. This has encouraged the development of non-VKA antagonist oral anticoagulants (NOACs)^1, 2^. Randomized controlled trials have investigated the safety and efficacy of the individual NOACs compared with VKA, finding these agents non-inferior to VKA with regard to efficacy, and associated with either equal or lower risk of major bleeding^5–9^. In Denmark, rivaroxaban was the first NOAC to be approved for the treatment of VTE (6^th^ of February 2012), followed by dabigatran (6^th^ of June 2014) and apixaban (31^st^ of July 2014). The American College of Chest Physicians treatment guidelines for VTE now recommend the initiation of a NOAC over VKA in patients without cancer, renal impairment, and liver disease^10^. Danish and European guidelines do not differentiate between the individual NOACs or VKA for the treatment of VTE^11^. Knowledge on the real-world initiation of NOACs for VTE is lacking and it is expected that the use of NOACs has increased since their approval and thus replacing the role of VKA. Little is known about which patients initiate rivaroxaban, apixaban or dabigatran versus VKA. The aims of this study were i) to examine temporal trends in initiation of the individual NOACs and VKA in VTE patients; ii) to assess potential differences in patient characteristics between those who initiate OAC treatment with one of the individual NOACs versus VKA.

## Results

**Figure 1:**
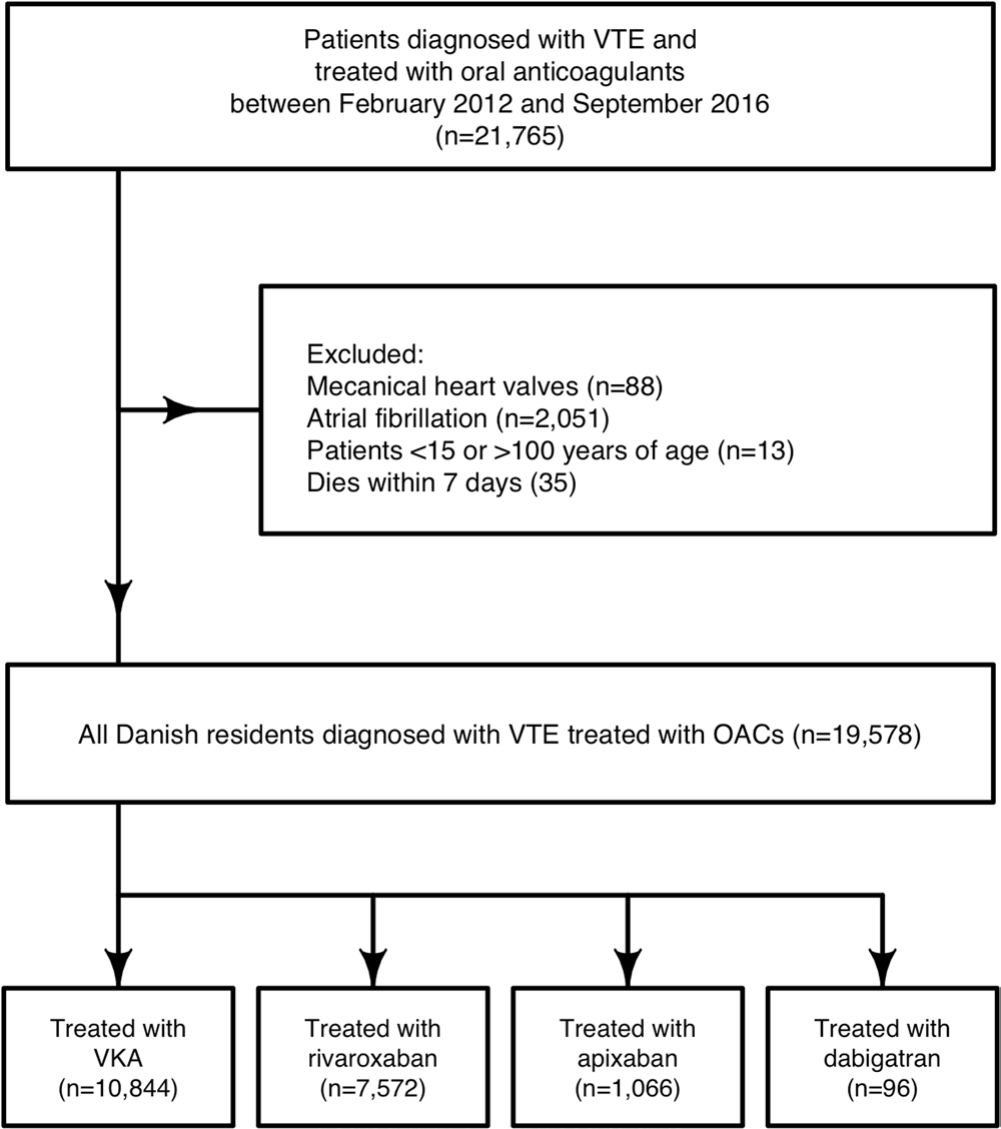
Selection of study cohort. Flow chart of selection process for the study population in the period between February 2012 and September 2016.

A total of 21,765 patients were eligible for inclusion (Fig. 1). After excluding patients, the study population comprised 19,578 patients of, which 10,844 (55.4%) were treated with VKA, 7,572 (38.7%) were treated with rivaroxaban, 1,066 (5.4%) were treated with apixaban, and 96 (0.5%) were treated with dabigatran (Table 1). In the study population a total of 11,772 (60.2%) patients had deep vein thrombosis (DVT) as inclusion event, and 7,806 (39.8%) patients had pulmonary embolism (PE) as inclusion event. Patients with DVT accounted for 61.9%, 59.9%, 44.2%, 52.1%, and 59.9% and in the VKA, rivaroxaban, apixaban, and dabigatran group respectively.

**Table 1:** Table of patient characteristics in apixaban, dabigatran, VKA and rivaroxaban groups.

	VKA (10,844)	Rivaroxaban (n = 7,572)	Apixaban (n = 1,066)	Dabigatran (96)
**Patient characteristic**				
Median age (IQR^+^)	66 [53–76]	66 [52–76]	69 [55–79]	71 [59–80]
Males (%)	5,775 (53.3)	4,109 (54.3)	532 (49.9)	45 (46.9)
**Index event** (**%**)				
PE	4,130 (38.1)	3,035 (40.1)	595 (55.8)	46 (47.9)
DVT	6,714 (61.9)	4,537 (59.9)	471 (44.2)	50 (52.1)
**Comorbidities** (**%**)				
Previous venous thromboembolism	1,973 (18.2)	911 (12.0)	101 (9.5)	10 (10.4)
Previous bleeding	1,099 (10.1)	691 (9.1)	111 (10.4)	9 (9.4)
Stroke	759 (7.0)	420 (5.5)	88 (8.3)	8 (8.3)
Acute myocardial infarction	426 (3.9)	205 (2.7)	51 (4.8)	<3
Ischemic heart disease	1,249 (11.5)	676 (8.9)	148 (13.9)	6 (6.2)
Peripheral vascular disease	307 (2.8)	148 (2.0)	23 (2.2)	3 (3.1)
Vascular disease	685 (6.3)	342 (4.5)	69 (6.5)	5 (5.2)
Chronic heart failure	509 (4.7)	332 (4.4)	49 (4.6)	4 (4.2)
Chronic kidney disease	550 (5.1)	188 (2.5)	38 (3.6)	0 (0.0)
Liver disease	224 (2.1)	112 (1.5)	21 (2.0)	<3
Hypertension	2,796 (25.8)	1,723 (22.8)	286 (26.8)	16 (16.7)
Diabetes mellitus	839 (7.7)	542 (7.2)	88 (8.3)	7 (7.3)
Cancer	1,499 (13.8)	844 (11.1)	134 (12.6)	15 (15.6)
COPD	1077 (9.9)	618 (8.2)	123 (11.5)	16 (16.7)
Thrombophilia §	211 (1.9)	71 (0.9)	4 (0.4)	0 (0.0)
Alloplastic surgery	205 (1.9)	169 (2.2)	23 (2.2)	<3
**Concomitant medication** (**%**)				
ADP-receptor blockers	518 (4.8)	366 (4.8)	84 (7.9)	10 (10.4)
Aspirin	1,814 (16.7)	1,016 (13.4)	167 (15.7)	10 (10.4)
Diuretics	2,436 (22.5)	1,509 (19.9)	250 (23.5)	18 (18.8)
Beta-blockers	1,649 (15.2)	969 (12.8)	169 (15.9)	8 (8.3)
Calcium channel blockers	1,701 (15.7)	1,023 (13.5)	177 (16.6)	10 (10.4)
Renin-angiotensin system blockers	3,055 (28.2)	1,939 (25.6)	310 (29.1)	21 (21.9)
Loop diuretics	1,417 (13.1)	785 (10.4)	140 (13.1)	16 (16.7)
Ulcer medication	2,555 (23.6)	1,625 (21.5)	284 (26.6)	24 (25.0)
Lipid modifying agents	2,188 (20.2)	1,420 (18.8)	232 (21.8)	14 (14.6)
Hormone replacement therapy	601 (5.5)	448 (5.9)	68 (6.4)	4 (4.2)
Oral contraceptives	547 (5.0)	367 (4.8)	41 (3.8)	<3
Non steroidal anti- inflammatory drugs	2,041 (18.8)	1,437 (19.0)	209 (19.6)	17 (17.7)

^+^IQR = Interquartile Range. ^§^Thrombophilia (factor v Leiden, prothrombin mutation, protein C, S, and antithrombin deficiency, antiphospholid syndrome and cardiolipin syndrome). PE: Pulmonary embolism. DVT: Deep venous thromboembolism. COPD: Chronic obstructive pulmonary disease.

### Temporal initiation

**Figure 2:**
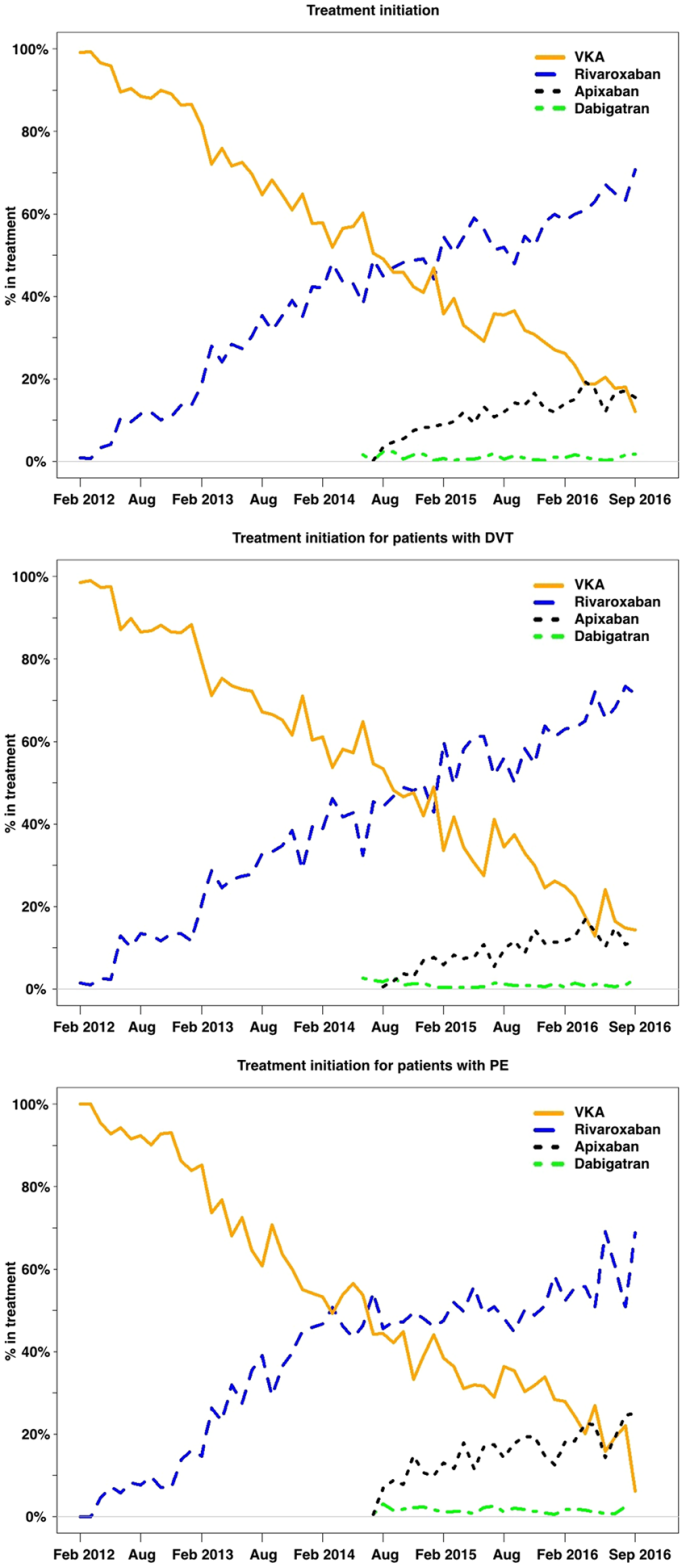
Temporal trends in initiation of anticoagulation therapy. Graph of temporal trend over initiation of oral anticoagulation therapy in patients with venous thromboembolism (VTE). Percentage of patients with VTE, pulmonary embolism (PE) or deep venous thromboembolism (DVT) initiating VKA, rivaroxaban, apixaban or dabigatran per month between February 2012 and September 2016. Rivaroxaban: p- value for increasing trend < 0.001, apixaban: p-value for increasing trend < 0.001, dabigatran: pValue for increasing trend < 0.991. VKA: p-value for decreasing trend < 0.001.

VKA was the preferred treatment at the beginning of the study period, with a steadily decline in the initiation until September 2016 (p-value for decreasing trend < 0001) (Fig. 2). An increase in the initiation of rivaroxaban was observed in the same period (p-value for increasing trend < 0001) (Fig. 2, and Supplementary Figure 1). By September 2016, 70% of patients with VTE were initiated on rivaroxaban vs. 12% initiated on VKA. From August 2014 to September 2016, the initiation of apixaban in patients with VTE increased from 1% to 16% (p-value for increasing trend < 0001). The use of dabigatran remained low during the whole study period, with 2% of patients with VTE initiated on dabigatran by September 2016 (Fig. 2). Similar observations were noted in patients with DVT or PE. By September 2016, 12%, 71%, 15% and 2% of patients diagnosed with DVT were initiated on VKA, rivaroxaban, apixaban and dabigatran respectively. Similarly 6%, 69% 25% and 0% of patients diagnosed with PE, were initiated on VKA, rivaroxaban, apixaban and dabigatran, respectively.

### Factors associated with initiation of a NOAC versus VKA

Table 2 shows factors associated with initiation of rivaroxaban, apixaban or dabigatran vs. VKA. Patients with previous VTE, chronic kidney disease, cancer, and thrombophilia were more likely to be initiated on VKA compared with either rivaroxaban or apixaban. However, because of the low number of patients in the dabigatran group, there were no significant differences in any factors associated with initiation of dabigatran vs. VKA. Patients with ischemic heart disease, liver disease, hypertension, previous stroke, and COPD were significantly more likely to be initiated on VKA compared with rivaroxaban. Patients older than 75, or patients with pulmonary embolism as index event, were significantly more likely to be initiated on apixaban compared with VKA. When dividing patient according to inclusion event (DVT or PE), similar factors were found to be associated with initiation of either one of the NOACs vs. VKA (Supplementary Tables 3 and 4). When the total NOAC group was compared with the VKA group, similar results were found in patients with VTE, DVT and PE (Supplementary Table 5).

**Table 2:** Factors associated with initiation of rivaroxaban, apixaban, or dabigatran, vs. VKA in patients with VTE.

variables	Odds ratios		
	Rivaroxaban vs. VKA	Apixaban vs. VKA	Dabigatran vs. VKA
**Sex**			
Female	Ref	Ref	Ref
Male	1.05 (0.98–1.12)	1.04 (0.90–1.21)	0.88 (0.58–1.33)
**Age groups**			
<65	Ref	Ref	Ref
65–75	1.01 (0.92–1.10)	0.99 (0.81–1.20)	1.42 (0.83–2.39)
>75	1.00 (0.92–1.09)	1.31 (1.08–1.58)*	1.61 (0.96–1.36)
**Index event**			
Deep venous thrombosis	Ref	Ref	Ref
Pulmonary embolism	1.05 (0.98–1.12)	1.68 (1.44–1.95)*	1.20 (0.79–1.82)
**Comorbidities**			
Previous VTE	0.78 (0.71–0.86)*	0.73 (0.57–0.93)*	0.68 (0.33–1.27)
Previous bleeding	1.01 (0.90–1.13)	0.92 (0.72–1.18)	0.82 (0.37–1.58)
Alloplastic surgery	1.22 (0.97–1.54)	1.03 (0.60–1.70)	0.97 (0.16–3.23)
Chronic kidney disease	0.42 (0.34–0.50)*	0.47 (0.32–0.69)*	NA
Liver disease	0.73 (0.57–0.95)*	0.93 (0.54–1.55)	NA
Heart failure	1.26 (1.06–1.50)	0.80 (0.55–1.16)	1.03 (0.30–2.74)
Ischemic heart disease	0.87 (0.75–0.99)*	1.21 (0.91–1.59)	0.45 (0.13–1.14)
Vascular disease	0.89 (0.70–1.13)	0.72 (0.40–1.22)	1.44 (0.34–4.05)
Hypertension	0.89 (0.82–0.97)*	0.93 (0.77–1.11)	0.48 (0.26–0.83)*
Acute myocardial infarction	0.85 (0.62–1.17)	1.49 (0.77–2.29)	0.76 (0.08–5.97)
Cancer	0.74 (0.67–0.82)*	0.76 (0.60–0.94)*	0.92 (0.50–1.60)
Diabetes mellitus	1.00 (0.88–1.14)	0.95 (0.71–1.24)	0.94 (0.39–1.97)
Stroke	0.86 (0.75–0.99)*	1.19 (0.90–1.56)	1.30 (0.57–2.61)
COPD	0.88 (0.78–0.99)*	1.08 (0.84–1.37)	1.80 (0.99–3.10)
Thrombophilia	0.58 (0.42–0.78)*	0.27 (0.08–0.69)*	1.75 (0.96–3.02)
Year (with 1 year increments)	2.21 (2.15–2.27)*	4.49 (4.14–4.89)*	2.76 (2.30–3.35)*

Odds ratio below 1 indicates in favor of VKA and above 1 in favor of rivaroxaban, apixaban or dabigatran. *Indicates significance.

## Discussion

This study investigated temporal trends in the initiation of oral anticoagulation therapy in patients with VTE. This study had two main findings: i) initiation of VKA declined whereas initiation of rivaroxaban and apixaban increased since their approval, while there were no temporal difference in the initiation of dabigatran. ii) Patients with chronic kidney disease, liver disease, thrombophilia, previous VTE, and cancer were more likely to be initiated on VKA compared with rivaroxaban or apixaban.

The cumbersome treatment with VKA has urged the development of NOACs, and results from this study showed that especially the initiation of rivaroxaban is being extensively implemented into clinical practice. Similar to this study, Badreldin *et al*.^12^ found in a cohort of VTE patients that only 2% of patients initiated dabigatran compared with 53%, 40%, and 5% initiated on rivaroxaban, apixaban, and edoxaban, respectively^12^. A plausible explanation for the low initiation of dabigatran could be that the treatment needs to be accompanied by parenteral heparin for at least 5 days^7^. In the RECOVER (Dabigatran versus warfarin in the treatment of acute venous thromboembolism) I and II trials, parenteral heparin was used for a median length of 7 days post randomization; hence evidence is lacking to support the use of dabigatran as mono-therapy without initial concomitant treatment with heparin^7, 9, 13^. On the contrary, rivaroxaban and apixaban were studied as mono-therapy in the EINSTEIN (Oral rivaroxaban for symptomatic venous thromboembolism and oral rivaroxaban for symptomatic pulmonary embolism) trial and in the AMPLIFY (Oral apixaban for the treatment of acute venous thromboembolism) trial^5, 6, 14^. Hence, the advantage of using dabigatran over either rivaroxaban or apixaban may be perceived as less apparent.

Results from this study showed that the odds of initiating treatment with NOACs in patients with cancer, renal impairment, and liver disease were significantly lower compared with VKA. The trials that established the safety and effectiveness of NOACs were not designed to investigate outcomes in patients with cancer, chronic kidney disease, or liver disease as most of these patients were excluded^5–7, 9, 13^. In our study, patients were classified as having had previous cancer that had occurred up to ten years prior to their diagnosis of VTE, thus there was no differentiation between active or previous cancer. In general, patients with active cancer are given parenteral anticoagulation therapy, sometimes as bridging to VKA^10, 11^. These patients have a poor prognosis, complicated by a high risk of recurrent VTE and high risk of bleeding^15–17^. The lower odds for initiating patients with cancer on a NOAC is likely explained by the perception from physicians that NOACs are not recommended in this patient group. Concerns have been raised about a possible increased risk of bleeding in patients with chronic kidney disease and liver failure, because of the pharmacokinetic profile of NOACs, which are contraindicated in patients with severe kidney disease (eGFR < 15 ml/min)^5–7, 9, 13^. The results from this study are in accordance with The American College of Chest Physician new guidelines from 2016, recommending initiation of a NOAC over VKA in patients without cancer, renal impairment, and liver disease^10^. The most recent guidelines from the European Society of Cardiology from 2014 do not differentiate between the choice of NOACs or VKA^11, 18^.

Interestingly, patients with indication for prolonged treatment, such as patients with previous VTE and thrombophilia were more likely to be initiated on a VKA compared with one of the NOACs^10, 11^. Even though the safety and efficacy of extended treatment with NOACs have been examined in randomized controlled trials, only dabigatran was directly compared with VKA in the REMEDY trial (Extended use of dabigatran, warfarin, or placebo in venous thromboembolism)^19^, while rivaroxaban and apixaban were compared with placebo in the EINSTEIN-EXT and AMPLIFY-EXT (Apixaban for extended treatment of venous thromboembolism)^5, 20^. As a result of this, Danish guidelines suggest that VKA should be used for extended treatment instead of NOACs. This also emphasizes the need for comparative safety and effectiveness studies to establish the risk-benefit profile of rivaroxaban and apixaban compared with VKA for extended treatment^21^.

The primary strength of this study is inherent in the use of the Danish nationwide registries, which have been associated with high internal validity, as the registration of diagnoses is considered reliable^22–24^. Several diagnoses have been validated through the registries, and relevant to this study the diagnosis of VTE has been associated with a positive predictive value between 75% and 90%^25, 26^. Another benefit is that the registries allow for the inclusion of all Danish residents and provide complete information regardless of socioeconomic status, geography, or participation in a health care insurance program, which increases the generalizability of results. Denmark has a reimbursement system for drugs, and therefore it is uncertain if the results from this study are representative for other countries. Despite the reimbursement system, NOACs still have a higher acquisition cost for the patient than VKA in Denmark, which is approximately three times higher for NOACs than for VKA. Unfortunately, we do not have information on socioeconomic or sociodemographic status in the utilized databases, which potentially are important factors for the initiation of a NOAC vs. VKA. Furthermore, there is a lack of important clinical variables that are not readily available through the registries, which includes: International normalized ratio, body mass index, smoking status, hepatic status, kidney function, and blood pressure. Since edoxaban was approved in Denmark subsequent to the study period, it was not possible to include this drug in the study.

In conclusion the initiation of NOACs, driven by rivaroxaban and apixaban, is increasing in patients with VTE, whereas the initiation of VKA is decreasing. By September 2016 12%, 70%, 16%, and 2% were initiated on VKA, rivaroxaban, apixaban, and dabigatran. Patients with previous VTE, chronic kidney disease, liver disease, cancer, and thrombophilia were significantly more likely to be initiated on VKAs compared with rivaroxaban or apixaban.

## Methods

### Data sources

In this Danish nationwide study data from three different registers were included: The Danish National Prescription Registry, the Danish National Patient Register, and The Danish Civil Registration System. All Danish residents are provided with a unique civil registration number, which enables individual-level linkage between the registries^22, 27^. The Danish Civil Registration System provides information on: vital status, gender, date of birth, and date of emigration and immigration^27^. The Danish National Patient Register provides information on all hospital admissions (in-patient and out-patient). Patients are registered with a primary diagnosis, being the primary reason for admission, and if necessary one or more secondary diagnoses. All hospital diagnoses in the register are classified according to the International Classification of Diseases, 10^th^ revision (ICD-10). All surgical procedures are registered according to the Nordic Classification of Surgical Procedures (NCPS)^23^. The Danish Registry of Medicinal Product Statistics provides information on all prescribed drugs redeemed at all Danish pharmacies, and includes information on strength, dosage, and date of redemption. All drugs are classified according to the Anatomical Therapeutic Chemical (ATC) classification system^22^.

### Study population

All patients diagnosed with VTE (in-patients and out-patients, primary and secondary diagnoses) in the period 6^th^ of February 2012 to 30^th^ of September 2016 were identified. Patients were included if they had redeemed a prescription of rivaroxaban, dabigatran, apixaban, or VKA 7 days prior or 7 days after the date of discharge with a VTE or date of diagnosis from an out-patient clinic, as has been performed previously^28^. Patients treated with VKA or rivaroxaban were included from the 6^th^ of February 2012, which was the date of approval of rivaroxaban. Patients treated with dabigatran or apixaban were included if they had redeemed a prescription after the 6^th^ of June 2014 or 31^st^ of July 2014, respectively, which were the dates of approval for these two drugs. Patients under 15 and over 100 years of age, and patients with atrial fibrillation were excluded. Furthermore, patients with mechanical heart valves were excluded.

### Comorbidities and concomitant medication

Comorbidities of interest were identified from the Danish National Patient Registry using ICD-10 diagnosis codes (Supplementary Tables 1 and 2). Comorbidities were registered within 10 years of a diagnosis with VTE. Baseline pharmacotherapy was identified through the Danish Registry of Medicinal Product Statistics using ATC-codes (Supplementary Tables 1 and 2). Redeemed prescriptions were identified within 180 days prior to the VTE diagnosis.

### Statistical analyses

Patient characteristics were presented as frequencies with percentages and as medians with interquartile ranges (IQR). Differences in baseline characteristics of included patients were investigated using the Chi-square test for discrete variables and Kruskal-Wallis rank sum test for continuous variables. Initiation of treatment with VKA rivaroxaban, apixaban, or dabigatran was plotted each month as the percentage of patients treated divided by the total number of patients initiating treatment. The temporal trends in initiation of rivaroxaban, apixaban, dabigatran and VKAs were investigated using the Cochran–Armitage test for trend. Multivariable logistic regression modeling, adjusted for calendar year, was used to investigate factors associated with the initiation of treatment with rivaroxaban, apixaban, and dabigatran vs. VKA. All statistical analyses were performed at a 0.05 significance level. Data management and statistical analysis were performed using SAS (Statistical Analytical System, version 9.4, SAS Institute, Gary, NC.) and R^29^.

### Ethics

Retrospective registry-based studies do not require approval from the Research Ethics Committee System. The Danish Data Protection Agency had approved use of data for this study (ref. no: 2007-58-0015/GEH-2014-012 I-Suite no: 02720).

## Electronic supplementary material

Supplementary
