# Supplementary material for: Temporal trends in initiation of VKA, rivaroxaban, apixaban and dabigatran for the treatment of venous thromboembolism - A Danish nationwide cohort study
Source: Sci Rep. 2017 Jun 13;7:3347. doi: 10.1038/s41598-017-03596-x (PMC5469820; doi:10.1038/s41598-017-03596-x)
Supplement: Supplementary file 1 — Supplementary [file 41598_2017_3596_MOESM1_ESM.pdf]

Temporal trends in initiation of VKA, rivaroxaban, apixaban and dabigatran for the treatment of venous thromboembolism - A Danish nationwide cohort study

Authors: Caroline Sindet-Pedersen M.Sc.* (1,2,3), Jannik Langtved Pallisgaard MD (1,2), Laila Staerk MD (1,2), Jeffrey S. Berger MD, MS (3), Morten Lamberts (1,4) Christian Torp-Pedersen MD, DMSc (1, 5), Gunnar H. Gislason MD, PhD (1,2,6, 7) Jonas Bjerring Olesen MD, PhD (1).

Affiliation

1) Department of Cardiology, Copenhagen University Hospital Herlev and Gentofte, 2900 Hellerup, Denmark.
2) Faculty of Health and Medical Sciences, University of Copenhagen, 2200 Copenhagen N, Denmark.
3) New York University School of Medicine, New York, New York, USA
4) Department of Cardiology, Copenhagen University Hospital Rigshospitalet, 2100 Copenhagen Ø, Denmark.
5) Department of Health, Science and Technology, Aalborg University, and Department of Epidemiology/Biostatistics and Cardiology, Aalborg University Hospital, Aalborg, Denmark.
6) The National Institute of Public Health, University of Southern Denmark, 1353 Odense, Denmark.
7) The Danish Heart Foundation, 1127 Copenhagen K, Copenhagen, Denmark.

*** Email for corresponding author**: carolinesindet@gmail.com

Supplementary

Table 1: List of ICD-10 diagnoses codes and ATC codes

Population		
Venous thromboembolism		
(Secondary and primary diagnoses codes, inpatient and outpatients)		
Pulmonary embolism	*Defined from diagnosis*	ICD-10: DI26
Deep venous thrombosis	*Defined from diagnosis*	ICD10: I801-I803, I808, I809, I821-I823, I828, I829
Rivaroxaban	*Defined from ATC*	B01AF01
Dabigatran	*Defined from ATC*	B01AE07
Apixaban	*Defined from ATC*	B01AF02
Vitamin K antagonists	*Defined from ATC*	B01AA03<br>B01AA04
**Comorbidities**		
(Secondary and primary diagnoses codes, inpatient and outpatients)		
Ischemic heart disease	*Defined from diagnosis*	ICD-10: DI20-25
Peripheral artery disease	*Defined from diagnosis*	ICD-10: DI70
Acute myocardial infarction	*Defined from diagnosis*	ICD-10: DI21, DI22
Stroke	*Defined from diagnosis*	ICD-10: DI63, DI64
Atrial fibrillation	*Defined from diagnosis*	ICD-10: DI48
Cancer	*Defined from diagnosis*	ICD-10: DC
Chronic kidney disease	*Defined from diagnosis*	ICD-10: DE102, DE112, DE132, DE142, DI120, DN02, DN03, DNO4, DN05, DN06, DN07, DN08, DN11, DN12, DN14, DN158, DN159, DN160, DN162 , DNI63, DN164, DN168, DN18, DN19, DN26, DQ61, DM300, DM313, DM319, DM321B
Hypertension	*Defined from combination treatment with at least two classes of antihypertensive drugs: adrenergic a-antagonists, non-loop diuretics, vasodilators, beta-blockers, calcium channel blockers and renin-angiotension system*	*ATC: C02A, C02B, C02C, C02L, C03A, C03B, C03D, C03E, C03X, C07B, C07C, C07D, C08G, C02DA, C09BA, C09DA, C02DB, C02DD, C02DG, C07A, C07B, C07C, C07D, C07F, C08, C09BB, C09DB, C09AA, C09BA, C09BB, C09CA, C09DA, C09DB, C09XA02, C09XA52*
Liver disease	*Defined from diagnosis*	DB15, DB16, DB17, DB18, DB19, DC22, DD684C, DK70, DK71, DK72, DK73, DK74, DK75, DK76, DK77, DZ944, DQ618A

Chronic obstructive pulmonary disease	*Defined from diagnosis*	ICD-10: DJ42, DJ43, DJ44
Chronic heart failure	*Defined from diagnosis*	ICD-10: DI110, DI42, DI50, DJ81
Thrombophilia	*Defined from diagnosis*	ICD-10: DD685, DD686
Diabetes mellitus	Diabetes mellitus Defined from glucose lowering drugs	ATC: A10
Mechanical heart valves or rheumatic mitral stenosis	*Defined from diagnosis and procedure codes*	ICD-10: DI050, DI060<br>NSCP: KFKD00, KFMD00, KFGE00, KFJF00
Alloplastic surgery	Defined from procedures	NSCP: KNFB, KNFC, KNGB, KNGC

Table 2: list of ATC-codes

ADP-receptor blockers	Defined from ATC-codes	B01AC except B01AC06/ B01AC56
Aspirin	Defined from treatment with acetylsalicylic acid	B01AC06, N02BA01, B01AC56
Ulcus medication	Defined from ATC-codes	A02
Lipid modifying drugs	Defined from ATC-codes	C10
Non steroidal anti-inflammatory drugs	Defined from ATC-codes	M01A
Contraceptives	Defined from treatment with hormonal contraceptives for systemic use	G03A
Hormone replacement therapy	Defined from treatment with estrogens, progestogens, androgens	G03C, G03D, G03E, GO3F
Diuretics	Defined from ATC-codes	C02L, C03A, C03B, C03D, C03E, C03X, C07C, C07D, C08G, C02DA, C09BA, C09DA, C09xa52
Loop	Defined from ATC-codes	C03C
Diuretics+ other antihypertensive drugs	Defined from ATC-codes	C02L
Beta- blocking agents	Defined from ATC-codes	C07A
Beta blocking agents combined with diuretics	Defined from ATC-codes	C07B , C07C, C07D
Beta blocking agents combined with calcium channel antagonists	Defined from ATC-codes	C07F
Calcium channel antagonists	Defined from ATC-codes	C08
Calcium channel antagonists and diuretics	Defined from ATC-codes	C08G
Renin angiotensin system	Defined from ATC-codes	C09XA02

inhibitors		
Renin angiotensin system inhibitors + diuretics	Defined from ATC-codes	C09BA, C09DA
Renin angiotensin system inhibitors + calcium channel blockers	Defined from ATC-codes	C09BB, C09DB

Table 3: Factors associated with initiation of rivaroxaban, apixaban, or dabigatran, vs. VKA in patients with deep venous thromboembolism. Odds ratio below 1 indicates in favor of VKA and above 1 in favor of rivaroxaban, apixaban or dabigatran

	Odds ratios		
	**Rivaroxaban vs. VKA**	**Apixaban vs. VKA**	**Dabigatran vs. VKA**
**Sex**			
Female	Ref	Ref	Ref
Male	1.02 (0.93-1.11)	1.11 (0.89-1.39)	0.92 (0.52-1.65)
**Age groups**			
<65	Ref	Ref	Ref
65-75	0.96 (0.86-1.07)	0.96 (0.86-1.07)	1.25 (0.58-2.58)
>75	1.00 (0.90-1.23)	1.00 (0.90-1.23)	1.66 (0.81-3.35)
**Index event**			
Deep venous thrombosis	NA	NA	NA
Pulmonary embolism	NA	NA	NA
**Comorbidities**			
Previous VTE	0.73 (0.64-0.82)*	0.71 (0.50-0.99)*	0.54 (0.18-1.26)
Previous bleeding	1.14 (0.97-1.24)	0.95 (0.63-1.40)	1.18 (0.43-2.73)
Alloplastic surgery	1.02 (0.75-1.40)	0.88 (0.35-1.96)	1.88 (0.29-6.61)
Chronic kidney disease	0.42 (0.32-0.54)*	0.63 (0.35-1.07)	NA
Liver disease	0.83 (0.60-1.48)	1.07 (0.48-2.19)	NA
Heart failure	1.50 (1.14-1.96)*	0.92 (0.46-1.76)	0.79 (0.04-4.36)
Ischemic heart disease	0.94 (0.77-1.14)	1.55 (0.98-2.39)	0.54 (0.08-1.92)
Vascular disease	0.89 (0.64-1.21)	1.17 (0.55-2.30)	1.83 (0.29-6.40)
Hypertension	0.86 (0.77-0.96)*	0.85 (0.63-1.22)	0.50 (0.19-1.07)
Acute myocardial infarction	0.73 (0.47-1.50)	0.49 (0.18-1.38)	NA
Cancer	0.71 (0.61-0.82)*	0.70 (0.49-0.99)*	0.77 (0.29-1.74)
Diabetes	1.16 (0.98-1.38)	1.33 (0.87-1.98)	0.95 (0.22-2.74)
Stroke	0.75 (0.62-0.91)*	1.04 (0.66-1.58)	1.54 (0.51-3.74)
COPD	0.78 (0.65-0.94)*	0.91 (0.57-1.41)	2.71 (0.85-4.84)
Thrombophilia	0.61 (0.43-0.86)*	0.17 (0.03-0.60)*	NA
Year (with 1 year increments)	2.28 (2.20-2.36)*	5.24 (4.62-5.98)	2.71 (2.12-3.53)

Table 4: Factors associated with initiation of rivaroxaban, apixaban, or dabigatran, vs. VKA in patients with pulmonary embolism Odds ratio below 1 indicates in favor of VKA and above 1 in favor of rivaroxaban, apixaban or dabigatran

	Odds ratios		
**Variables**	**Rivaroxaban vs. VKA**	**Apixaban vs. VKA**	**Dabigatran vs. VKA**
**Sex**			
Male	Ref	Ref	Ref
Female	1.09 (0.98-1.22)	0.97 (0.80-1.20)	0.88 (0.48-1.62)
**Age groups**			
<65	Ref	Ref	Ref
65-75	1.06 (0.93-1.22)	1.63 (1.26-2.11)	1.63 (0.75-3.53)
>75	1.02 (0.89-1.17)	1.06 (0.80-1.40)	1.64 (0.77-3.55)
**Index event**			
Deep venous thrombosis	NA	NA	NA
Pulmonary embolism	NA	NA	NA
**Comorbidities**			
Previous VTE	0.87 (0.74-1.02)	0.75 (0.52-1.05)	0.87 (0.29-2.11)
Previous bleeding	0.87 (0.74-1.03	0.92 (0.67-1.25)	0.87 (0.15-1.40)
Alloplastic surgery	1.51 (1.06-2.16)	1.13 (0.56-2-15)	NA
Chronic kidney disease	0.41 (0.31-0.54)	0.37 (0.21-0.62)*	NA
Liver disease	0.61 (0.39-0.93)	0.85 (0.39-1-72)	NA
Heart failure	1.12 (0.90-1.40)	0.75 (0.47-1.17)	1.10 (0.24-3.51)
Ischemic heart disease	0.81 (0.67-0.98)	1.04 (0.73-1.47)	0.42 (0.06-1.47)
Vascular disease	0.93 (0.65-1.32)	0.43 (0.17-0.97)*	1.00 (0.05-5.06)
Hypertension	0.93 (0.82-1.05)	0.99 (0.78-1.25)	0.47 (0.21-1.00)
Acute myocardial infarction	0.93 (0.60-1.46)	3.24 (1.27-9.17)*	2.06 (0.15-5.75)
Cancer	0.78 (0.67-0.90)	0.79 (0.59-1.04)	1.08 (0.48-2.22)
Diabetes	0.83 (0.68-1.02)	0.76 (0.51-1.10)	0.94 (0.27-2.49)
Stroke	1.00 (0.82-1.24)	1.32 (0.91-1.90)	1.05 (0.25-3.03)
COPD	0.96 (0.82-1.12)	1.16 (0.86-1.53)	1.48 (0.65-3.05)
Thrombophilia	0.51 (0.26-0.94)	0.50 (0.08-1.88)	NA
Year (with 1 year increments)	2.12 (2.03-2.22)	4.01 (3.60-4.49)	2.79 (2.14-3.75)

Table 5: Factors associated with initiation of NOACs vs. VKA in patients with venous thromboembolism, Deep venous thromboembolism or pulmonary embolism

	NOAC vs VKA in patients with VTE	NOAC vs VKA in patients with DVT	NOAC vs VKA in patients with PE
**Sex**			
Female	Ref		
Male	1.05 (0.98-1.12)	1.02 (0.93-1.11)	1.07 (0.97-1.19)
**Age groups**			
<65	Ref		
65-75	1.01 (0.92-1.10)	1.01 (0.90-1-13)	1.07 (0.94-1.24)
>75	1.00 (0.92-1.09)	0.97 (0.89-1.01)	1.09 (0.95-1.22)
**Index event**			
Deep venous thrombosis	Ref	NA	NA
Pulmonary embolism	1.10 (1.03-1.18)*	NA	NA
**Comorbidities**			
Previous VTE	0.78 (0.71-0.85)*	0.73 (0.64-0.82)*	0.86 (0.74-1.00)
Previous bleeding	1.00 (0.90-1.12)	1.14 (0.98-1.33)	0.87 (0.74-1.02)
Alloplastic surgery	1.22 (0.97-1.53)	1.03 (0.76-1.40)	1.48 (1.05-2.10)*
Chronic kidney disease	0.42 (0.35-0.51)*	0.44 (0.34-0.56)*	0.41 (0.31-0.53)*
Liver disease	0.75 (0.59-0.97)*	0.84 (0.61-1.15)	0.65 (0.43-0.97)*
Heart failure	1.22 (1.03-1.44)*	1.45 (1.11-1.89)*	1.08 (0.87-1.34)
Ischemic heart disease	0.90 (0.78-1.02)	0.98 (0.81-1.19)	0.84 (0.70-1.01)
Vascular disease	0.88 (0.70-1.11)	0.93 (0.68-1.26)	0.85 (0.60-1.20)
Hypertension	0.89 (0.82-0.97)*	0.85 (0.76-0.95)*	0.93 (0.83-1.05)
Acute myocardial infarction	0.91 (0.67-1.23)	0.69 (0.45-1.07)	1.11 (0.72-1.72)
Cancer	0.75 (0.68-0.82)*	0.71 (0.62-0.81)*	0.78 (0.67-0.90)
Diabetes	1.01 (0.89-1.14)	1.17 (0.99-1.40)	0.89 (0.69-1.12)
Stroke	0.90 (0.78-1.03)	0.78 (0.65-0.94)*	1.05 (0.86-1.24)
COPD	0.91 (0.81-1.03)	0.82 (0.68-0.98)*	0.99 (0.85-1.15)
Thrombophilia	0.56 (0.41-0.75)*	0.58 (0.41-0.81)*	0.50 (0-26-0.95)*
Year (with 1 year increments)	2.37 (2.31-2.44)*	2.41 (2.32-2.50)*	2.33 (2.23-2.44)*

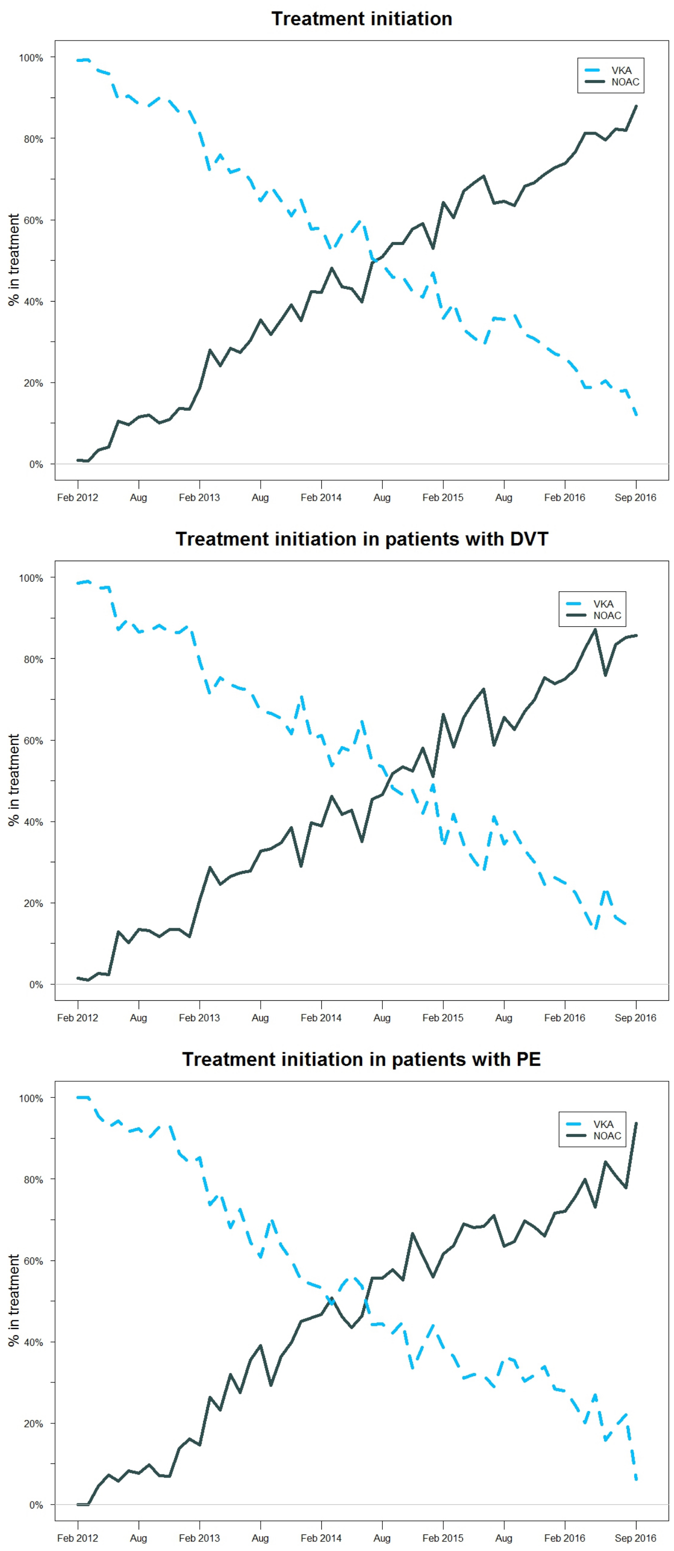

Figure 1: Graph of temporal trend over initiation of oral anticoagulation therapy in patients with venous thromboembolism (VTE). Percentage per month of patients with VTE, pulmonary embolism (PE) or deep venous thromboembolism (DVT) initiating VKA or NOAC, between February 2012 and September 2016**.** VKA: p- value for decreasing trend < 0.001, NOAC: p-value for increasing trend < 0.001.
